# A Volume Challenge Reveals the Diagnosis of Pediatric Restrictive Cardiomyopathy

**DOI:** 10.1155/2022/4707309

**Published:** 2022-08-17

**Authors:** Elyssa Cohen, Conor P. O'Halloran, Philip T. Thrush, T. Marsha Ma, Paul Tannous

**Affiliations:** ^1^Ann & Robert H. Lurie Children's Hospital of Chicago, Chicago, IL, USA; ^2^Northwestern University Feinberg School of Medicine, Chicago, IL, USA; ^3^Loyola University Medical Center, Chicago, IL, USA

## Abstract

A healthy 11-year-old girl presented with exercise intolerance of unclear etiology, and her physical exam was notable for a 3/6 systolic ejection murmur at the left upper sternal border with radiation to the back. Extensive noninvasive workup consisted of ECG, transthoracic echocardiogram, and cardiac MRI/MRA, which were all nondiagnostic. She was ultimately referred for cardiac catheterization. Baseline invasive hemodynamics demonstrated a normal cardiac index and pulmonary vascular resistance but was notable for mildly elevated right and left end-diastolic pressures. A diagnosis remained elusive, so a 500 mL volume challenge was performed, which unmasked right and left ventricular waveform transformations to reveal the pathognomonic “square root sign” of restrictive cardiomyopathy with concordant RV/LV respirophasic variation. These findings and her clinical history allowed for the rare pediatric diagnosis of restrictive cardiomyopathy early in her clinical course, prior to the development of overt signs of pathologic myocardial remodeling, such as pulmonary hypertension and biatrial enlargement.

## 1. Case Report

An 11-year-old healthy female presented to her pediatrician for a well-child check with the new complaint of not being able to keep up with her peers during soccer due to shortness of breath and pain characterized as a shooting sensation down both legs. Physical exam was notable for a 3/6 systolic ejection murmur at the left upper sternal border with radiation to the back, mildly diminished lower extremity pulses, and normal four extremity blood pressures.

**Figure 1:**
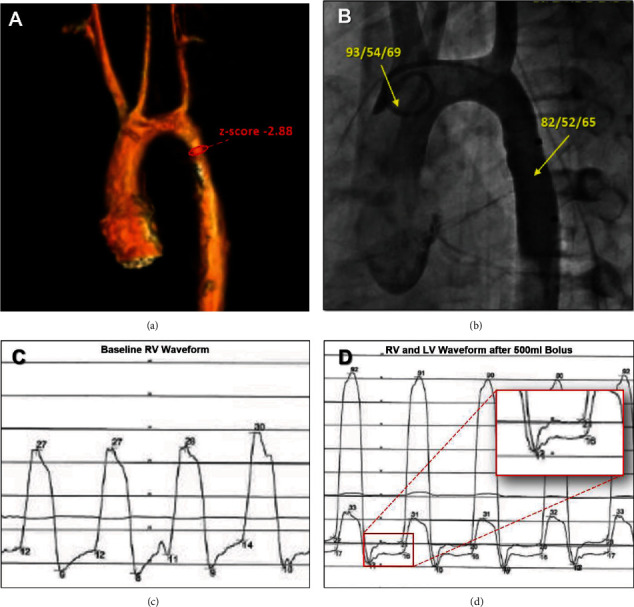
Cardiac MRA, aortic arch angiography, and hemodynamic waveforms before and after volume challenge. (a) Cardiac MRA showed a crenellated arch with hypoplasia of the proximal descending thoracic aorta (*z*-score of –2.88), but no discrete coarctation. (b) Catheterization was performed and confirmed no arch gradient. (c) Pressure tracings document an elevated RVEDp (12 mmHg) and LVEDp (16 mmHg) but with grossly normal diastolic waveforms at the start of the case. (d) After a 500 mL bolus, RVEDp and LVEDp increased to 16 and 20 mmHg, respectively, and dramatic change in the diastolic waveforms was observed with unmasking of a “classic” square root sign and concordant RV/LV respirophasic variation.

An electrocardiogram was performed and demonstrated normal sinus rhythm, no atrial enlargement, no right or left ventricular hypertrophy, and no evidence of right or left ventricle strain. Transthoracic echocardiogram documented flow acceleration across the aortic arch with a peak velocity of 4 m/sec, normal systolic function with an ejection fraction of 70%, normal atrial and ventricular size, and no evidence to suggest decreased compliance. Cardiac MRI/MRA was ordered to further delineate anatomy, and it showed a crenellated aortic arch with mild hypoplasia of the proximal descending thoracic aorta (*z*-score of -2.88) with a peak velocity of 2.1 m/s, but no discrete coarctation (Figure 1(a)). There were no abdominal aortic lesions or evidence of endocardial fibroelastosis. Given the equivocal nature of data thus far collected and no clear etiology for her exertional symptoms, a cardiac catheterization was performed. Baseline hemodynamics revealed a normal cardiac index of 4.6 L/min/m^2^, normal PVR of 1.7 iWU, no arch gradient (Figure 1(b)), mildly elevated RVEDp (12 mmHg) and LVEDp (16 mmHg) with normal diastolic waveforms (Figure 1(c)). Collectively, these data did not provide a unifying diagnosis and the etiology for her exercise intolerance remained uncertain.

To further investigate the significance of her elevated end-diastolic pressures, a 500 mL normal saline bolus was administered, acutely increasing the RVEDp and LVEDp to 16 and 20 mmHg, respectively. In addition to the rise in end-diastolic pressure, we observed a dramatic change in the diastolic waveform with unmasking of a “classic” square root sign and concordant RV/LV respirophasic variation (Figure 1(d)). Four-chamber end-diastolic pressures did not equalize as would be seen in constrictive pericardial disease. Collectively, these findings and her clinical history were most consistent with a diagnosis of restrictive cardiomyopathy (RCM). Arch intervention was not indicated, and she was referred to heart failure clinic for medical management.

As an outpatient at heart failure clinic, she was started on 10 mg of Lasix daily for symptom prevention. She had an unremarkable Holter monitor and exercise stress test. Genetic testing has not identified any known cardiomyopathy-associated genes to explain her restrictive phenotype.

Now two years following diagnosis, she is asymptomatic on a daily diuretic. Interval hemodynamic evaluations and echocardiograms demonstrate preserved systolic function and no evidence of pulmonary hypertension. She continues to enjoy being active and participating on her soccer team at a competitive level.

## 2. Discussion

Restrictive cardiomyopathy is a rare diagnosis in the pediatric population with an incidence of 0.03-0.04 cases per 100,000 children [1, 2]. Patients often present with a history of chronic and subtle progression of symptoms. Diagnosis is typically made later in the disease course than it was for this patient, usually when there are overt signs of pathologic myocardial remodeling such as biatrial enlargement, hepatomegaly, pulmonary hypertension, and pathognomonic changes in intracardiac pressure waveforms [3–5].

This patient presented early with exertional symptoms, and it was her nonspecific physical exam findings that led to the investigation for a cardiac etiology. Prior to cardiac catheterization, she had undergone an extensive workup with an echocardiogram and cardiac MRI/MRA, which were only significant for a mildly hypoplastic distal thoracic aortic arch—insufficient in explaining her symptoms. During her hemodynamic evaluation in the catheterization lab, her end-diastolic ventricular pressures were observed to be mildly elevated; however, the ventricular diastolic waveforms were not consistent with restrictive physiology or constrictive pericarditis. Considering the patient's NPO status and possibility that she was presenting with mild hypovolemia, a 500 mL bolus was administered to replete her volume status. Immediately, the RVEDp and LVEDp increased, the RV and LV diastolic waveforms transformed to reveal the “square root sign,” and the ventricular pressures demonstrated concordant respirophasic variation, findings consistent with restrictive cardiomyopathy [6–8]. Thus, her symptoms are most likely secondary to left atrial hypertension exacerbated by peak exercise.

In conclusion, cardiac etiologies should be considered in patients with unexplained exercise intolerance, particularly in the setting of abnormal cardiovascular examination. Dehydration caused by routine fasting before cardiac catheterization may mask the characteristic hemodynamic findings of RCM. Careful fluid administration can unmask these findings, yielding a definitive diagnosis in otherwise unclear cases.
